# An update on clinical oncology for the non-oncologist

**DOI:** 10.1590/S1679-45082016MD3550

**Published:** 2016

**Authors:** Rafael Aliosha Kaliks

**Affiliations:** 1 Hospital Israelita Albert Einstein, São Paulo, SP, Brazil.

**Keywords:** Neoplasms/trends, Immunotherapy/trends

## Abstract

Recent advances in the understanding of tumor driver mutations, signaling pathways that lead to tumor progression, and the better understanding of the interaction between tumor cells and the immune system are revolutionizing cancer treatment. The pace at which new treatments are approved and the prices at which they are set have made it even more difficult to offer these treatments in countries like Brazil. In this review we present for the non-oncologist these new treatments and compare their availability in Brazilian public health system and private health system with that of developed countries.

## RESUMO

Avanços recentes na compreensão de mutações promotoras de desenvolvimento do câncer, sinalização que leva à progressão de tumores, e o avanço no entendimento da interação entre as células tumorais e o sistema imunológico estão revolucionando o tratamento do câncer. A velocidade com que novos tratamentos são aprovados e o alto custo das medicações dificultam a disponibilização de terapêuticas em países como o Brasil. Nesta revisão, apresentamos ao não oncologista esses novos tratamentos e comparamos sua disponibilidade nos sistemas público e privado de saúde no Brasil com os países desenvolvidos.

## INTRODUCTION

Within the last few years, the field of systemic therapy in medical oncology has seen two dramatic changes. First, the advances in the understanding of genetic abnormalities led to the discovery of various tumor driver mutations with the consequent development of different targeted therapies. Second, the better understanding of interaction between tumor cells and immune system led to the now much broader field of immuno-oncology and the consequent development of immunotherapies, which is currently being tested for treatment of different cancer types. In addition to these advances, a few new traditional chemotherapies have been approved and some already well-known treatments have had their indication broadened. Our objective is to review, for non-oncologists, the most recent advances of modern systemic cancer treatment.

## TARGETED THERAPIES

New technologies developed after the year 2000 allowed progressively more ambitious and thorough evaluation of tumors at molecular level. A major initiative has been the whole genome sequencing of various tumors, launched in 2005 as part of The Cancer Genome Atlas.^(1)^ So far, more than 20 different tumor types have been fully sequenced. The advances in the evaluation of genetic and epigenetic abnormalities and their consequences on the transcriptome have allowed a better understanding and further mapping of an intricate signaling pathway network, which characterizes the hallmarks of cancer cells.^(2,3)^ The identification of driver mutations have crossed histologically driven tumor classifications, leading to a new way of looking at tumors, now based on the mutations rather than histology or organ in which the tumor arises. Although the identification of targets in tumor cells led to the development of various targeted therapies, we are now confronting the fact that these treatments have unfortunately not led to cure in metastatic cancers as once expected, despite the fact that outcomes such as progression free survival and overall survival have improved. In addition, before choosing the therapies, it is necessary to test the target, which sometimes requires sophisticated and costly techniques. Considering that some targets may be present in less than 5 to 10% of the patient population, many of them need to be tested in order to find one eligible for the targeted treatment. Adding the costs of tests and the drugs, these therapies are almost prohibitive for the Brazilian Public Health System, which prevent a significant majority of our population from receiving such treatments.

Table 1 shows selected new targeted therapies made available in the last three years. Approval in Brazil has been limited, mainly due to regulatory delays, but certainly influenced by costs as well. The table describes the main indications, the targets for each drug and the most important outcomes reported in clinical trials.

**Table 1:** Targeted therapies

Reference	Tumor type (by organ)	Name of drug	Mechanism of action	Indication	Main results	Availability in Brazil
Verma et al.^(4)^	Breast	Ado-trastuzumab emtansine (T-DM1)	Antibody-drug conjugate against Her2+ cells	Metastatic Her2+ breast cancer, after failing trastuzumab and taxane	Improved PFS and overall survival compared with lapatinib and capecitabine	Registered in Brazil. Not available in Brazilian Public Health System
Swain et al.^(5)^	Breast	Pertuzumab	Her2 inhibition	Metastatic Her2+ breast cancer	Improved PFS and overall survival compared with trastuzumab and taxane	Registered in Brazil. Not available in Brazilian Public Health System
Piccart et al.^(6)^	Breast	Everolimus	mTOR inhibitor	Metastatic HR+ and Her2- breast cancer in combination with exemestane	Improved PFS compared with second line exemestane alone	Registered in Brazil. Not available in Brazilian Public Health System
Turner et al.^(7)^	Breast	Palbociclib	CDK4 and CDK6 inhibitor	Metastatic HR+ and Her2- breast cancer in combination with fulvestrant	Improved PFS compared with second line Fulvestrant alone	Not registered in Brazil
Tewari et al.^(8)^	Cervix	Bevacizumab	VEGF inhibitor	Metastatic cervical cancer	Improved overall survival when added to chemotherapy	Registered in Brazil. Not available in the Brazilian Public Health System
Grothey et al.^(9)^	Colorectal	Regorafenib	Multikinase inhibitor	Previously treated metastatic colorectal cancer	Modest improvement in overall survival compared with supportive care alone	Not registered in Brazil
Fuchs et al.^(10)^	Gastric	Ramucirumab	VEGFR2 antagonist	Inoperable gastric or gastroesophageal junction adenocarcinoma after prior chemotherapy	Improved survival compared with placebo	Not registered in Brazil
Demetri et al.^(11)^	GIST	Regorafenib	Multikinase inhibitor	Metastatic GIST after standard treatment with imatinib and sunitinib	Improved PFS compared with placebo	Not registered in Brazil
Wu et al.^(12)^; Sequist et al.^(13)^	Lung	Afatinib	EGFR inhibitor	Metastatic NSCLC with EGFR exon 19 deletion or L858R EGFR mutation	Improved PFS compared with gemcitabine and cisplatin or with cisplatin and pemetrexed	Not registered in Brazil
Shaw et al.^(14,15)^	Lung	Crizotinib	ALK inhibitor, ROS1 inhibitor	Metastatic NSCLC with ALK-EML4 fusion, or with ROS1 rearrangement	Improved PFS compared with pemetrexed in platinum refractory disease	Not registered in Brazil
Shaw et al.^(16)^	Lung	Ceritinib	ALK inhibitor	Metastatic ALK-rearranged NSCLC	Responses in naïve and crizotinib pretreated disease	Not registered in Brazil
Chapman et al.^(17)^	Melanoma	Vemurafenib	BRAF inhibitor	Metastatic melanoma with BRAF V600E mutation	Improved overall survival and PFS compared with Dacarbazine	Registered in Brazil. Not available in the Brazilian Public Health System
Robert et al.^(18)^	Melanoma	Dabrafenib	BRAF inhibitor	Metastatic melanoma BRAF V600E mutation	Improved overall survival when combined with Trametinib, compared with Vemurafenib	Not registered in Brazil.
Robert et al.^(18)^	Melanoma	Trametinib	MEK inhibitor	Metastatic Melanoma with BRAF V600E or V600K mutation	Improved overall survival when combined with Dabrafenib, compared with Vemurafenib	Not registered in Brazil
Ledermann et al.^(19)^	Ovary	Olaparib	Inhibitor of poly (ADP-ribose) polymerase	BRCA mutated advanced ovarian cancer	Improved PFS compared with placebo in platinum sensitive relapse	Not registered in Brazil
Brose et al.^(20)^	Thyroid	Sorafenib	Multi-kinase inhibitor	Metastatic differentiated thyroid cancer refractory to radioactive iodine	Improved PFS compared with placebo	Not registered in Brazil for this indication
Schlumberger et al.^(21)^	Thyroid	Lenvatinib	VEGF receptor inhibitor, PDGFR inhibitor, RET and KIT	Metastatic differentiated thyroid cancer refractory to radioactive iodine	Improved PFS compared with placebo	Not registered in Brazil
Elisei et al.^(22)^	Medullary thyroid	Cabozantinib	MET, VEGFR2 and RET inhibitor	Progressive metastatic medullary thyroid cancer	Improved PFS compared with placebo	Not registered in Brazil
Wells et al.^(23)^	Medullary thyroid	Vandetanib	RET kinase inhibitor and VEGF inhibitor	Progressive metastatic medullary thyroid cancer	Improved PFS compared with placebo	Not registered in Brazil

T-DM1: Kadcyla; Her2: human epidermal growth factor receptor 2; PFS: progression-free survival; CDK: cyclin-dependent kinase; VEGF: vascular endothelial growth factor; VEGFR: vascular endothelial growth factor receptor; GIST: gastrointestinal stromal tumor; EGFR: epidermal growth factor receptor; NSCLC: non small cell lung cancer; ALK: anaplastic lymphoma kinase; ROS1: ROS proto-oncogene 1; BRAF: proto-oncogene B-Raf; MEK: mitogen activated protein kinase; BRCA: breast cancer gene; PDGFR: platelet derived growth factor receptor; MET: hepatocyte growth factor receptor; RET: rearranged during transfection; KIT: proto-oncogene c-Kit.

Some targeted therapies that have been approved and made available in the private health system in Brazil for several years still have limited access in the Brazilian Public Health System. Some examples are trastuzumab for metastatic Her2+ breast cancer; erlotinib and gefitinib for epidermal growth factor receptor (EGFR) mutated metastatic lung cancer; cetuximab and panitumumab for RAS-wild type metastatic colorectal cancer, in addition to several treatments used in hematological malignancies, which are not the focus of this report. Some other targeted treatments have been available for several years in North America and/or Europe, but they are still not registered in Brazil. Examples are aflibercept for colorectal cancer, pazopanib and trabectedine for soft tissue sarcomas, and axitinib for renal cell carcinoma.

## IMMUNOTHERAPY

Human immune system has been known for quite some time for recognizing tumor antigens and mounting an immune response, although the actual explanation for the variability in tumor control by the immune system remains elusive. Cancer cells are capable of evading the immune surveillance by suppressing tumor-directed immunity through mechanisms described over the last two decades.^(24)^ It occurs by inhibiting helper and cytotoxic T cells while stimulating regulatory T cells instead. Inhibitory mechanisms determined by cytotoxic T-lymphocyte-associated antigen-4 (CTLA-4), programmed death 1 (PD1) and its ligand programmed death ligand 1 (PD-L1) can currently be targeted and inhibited by new immunotherapies, which lead to unblocking the immune response. This will ultimately unleash an immune attack on cancer cells. Anti-CTLA-4 antibodies as well as PD1 and PD-L1 inhibitors are already approved and used to treat a limited number of tumor types (melanoma and lung cancer), and promising preliminary results indicate potential future use in a large variety of cancers.

Table 2 outlines the new immunotherapies, its approved indications, mechanism of action and main results in clinical trials.

**Table 2:** Immunotherapies

Reference	Tumor type (by organ)	Name of drug	Mechanism of action	Indication	Main results	Availability in Brazil
Hodi et al.^(25)^; Robert et al.^(26)^	Melanoma	Ipilimumab	Anti-CTLA-4	Metastatic melanoma	Improved overall survival compared with gp100 vaccine and improved overall survival when added to dacarbazine compared with dacarbazine alone	Registered in Brazil. Not available in the Brazilian Public Health System
Robert et al.^(27)^ e Weber et al.^(28)^	Melanoma	Nivolumab	Anti-PD1	Metastatic melanoma without BRAF mutation or after progression on Ipilimumab and BRAF inhibitor	Improved overall survival and progression free survival compared with dacarbazine	Not registered in Brazil
Robert et al.^(29)^	Melanoma	Pembrolizumab	Anti-PD1	Metastatic melanoma	Improved overall survival and progression free survival compared with ipilimumab	Not registered in Brazil
Brahmer et al.^(30)^	Lung	Nivolumab	Anti-PD1	Metastatic NSCLC, squamous histology	Improved overall survival, progression free survival and response rate compared with Docetaxel	Not registered in Brazil
Garon et al.^(31)^	Lung	Pembrolizumab	Anti-PD1	Metastatic NSCLC	Significant antitumor activity	Not registered in Brazil

CTLA-4: cytotoxic T-lymphocyte-associated antigen-4; PD1: programmed death 1; BRAF: proto-oncogene B-Raf; NSCLC: non small cell lung cancer.

The successful combination of two immunotherapies was already reported. Combined nivolumab and ipilimumab had better results than either drug alone to treat metastatic melanoma.^(32,33)^ Both nivolumab and pembrolizumab, as well as other anti-PD1, anti PD-L1 and combinations with anti-CTLA-4, are under test for a variety of tumors, with some extraordinary preliminary results. Positive results with these immunotherapies have been reported in kidney, bladder, pancreatic, metastatic colorectal cancer related to Lynch syndrome, gastroesophageal cancer and glioblastoma, among others. Of note, although, is that for most diseases exist a clear correlation of benefit with the higher expression of PD-L1 on tumor cells,^(34,35)^ and there is still no standardized evaluation for the expression of PD1 or PD-L1. An unique aspect related to immunotherapies is sometimes the significant delayed response, which has been reported both with anti-CTLA-4 as well as anti- -PD1 inhibitors.^(36,37)^ This highlights the need for careful consideration before deeming these drugs ineffective, and it has led to the establishment of a different set of response criteria, known as immune-related response criteria (irRC).^(38)^ Immune related adverse events derive from the activation of autoimmune-mediated diseases in the skin, gastrointestinal tract, liver and endocrine system. The most clinically relevant adverse event is diarrhea, which may have late onset and be life threatening if not rapidly and properly treated.

## OTHER NEW SYSTEMIC TREATMENTS

In addition to the new targeted therapies and immunotherapies, few other new treatments (with various mechanisms of action) with significant clinical impact have emerged and been approved for clinical use in recent years.

Table 3 describes new systemic treatments, its indications, mechanisms of action and main results in clinical trials.

**Table 3:** Other new cancer therapies

Reference	Tumor type (by organ)	Name of drug	Mechanism of action	Indication	Main results	Availability in Brazil
Ryan et al.^(39)^	Prostate	Abiraterone	Blocks cytochrome P450 17 alpha-hydroxilase reducing androgen production	Metastatic castration resistant prostate cancer	Improvement in overall survival compared with prednisone	Registered in Brazil. Not available in the Brazilian Public Health System
Beer et al.^(40)^	Prostate	Enzalutamide	Androgen receptor blocker and androgen receptor signal inhibitor	Metastatic Castration resistant prostate cancer	Improvement in overall survival compared with placebo	Registered in Brazil for castration and chemotherapy refractory disease. Not available in Brazilian Public Health System
Sweeney et al.^(41)^; James et al.^(42)^	Prostate	Docetaxel	Interferes with mitotic spindle	Upfront treatment of castration sensitive metastatic prostate cancer	Improvement in overall survival when added to castration, compared with castration alone	Registered in Brazil. Available in the Brazilian Public Health System
Parker et al.^(43)^	Prostate	Rad 223 dichloride	Alpha emitter that targets bone metastases	Metastatic (to the bones) castration resistant prostate cancer	Improved overall survival compared with placebo	Registered in Brazil. Not available in the Brazilian Public Health System
Cortes et al.^(44)^	Breast	Eribulin mesilate	Microtubule inhibitor	Previously treated metastatic breast cancer	Improved overall survival compared with treatment of physicians choice	Registered in Brazil. Not available in the Brazilian Public Health System

There is currently a very vivid discussion around the world about the significant costs associated with new cancer therapy in general, and specifically about anti-cancer drugs. Immunotherapies, which seem to be on their way to become indicated for a large proportion of cancer patients, and some of the newer targeted therapies can cost hundreds of thousands of dollars per patient annually.^(45)^ Cost is certainly a significant limiting factor for these drugs becomes available in Brazil.

Some good cancer treatments are still under registration process in Brazil, highlighting the gap between what is practice here in comparison with developed countries. No less important is the significant difference between what is registered and used in the private health system and what is available and used in the Brazilian public health system. Unless pricing of drugs becomes more reasonable in the near future, and unless health technology evaluation for the public health system starts to be dictated by well-established standards and pre-specified cost-effectiveness limits, new cancer therapies will be ever more limited in developing countries like Brazil, and as a consequence the difference between what is practiced internationally and in our country will widen significantly.
